# Gold-Catalyzed Homogeneous (Cyclo)Isomerization Reactions

**DOI:** 10.3389/fchem.2019.00296

**Published:** 2019-05-14

**Authors:** Marta Marín-Luna, Olalla Nieto Faza, Carlos Silva López

**Affiliations:** Departamento de Química Orgánica, Universidade de Vigo, Vigo, Spain

**Keywords:** gold, catalysis, isomerization, allenes, 1, *n*-enynes, 1, *n*-diynes

## Abstract

Gold is currently one of the most used metals in organometallic catalysis. The ability of gold to activate unsaturated groups in different modes, together with its tolerance to a wide range of functional groups and reaction conditions, turns gold-based complexes into efficient and highly sought after catalysts. Natural products and relevant compounds with biological and pharmaceutical activity are often characterized by complex molecular structures. (Cyclo)isomerization reactions are often a useful strategy for the generation of this molecular complexity from synthetically accessible reactants. In this review, we collect the most recent contributions in which gold(I)- and/or gold(III)-catalysts mediate intramolecular (cyclo)isomerization transformations of unsaturated species, which commonly feature allene or alkyne motifs, and organize them depending on the substrate and the reaction type.

## 1. Introduction

Historically, most civilizations have considered gold as synonym of power, purity, beauty and wealth. In contrast, for a long time, gold was chemically ignored and misconceived as an inert element. It was only at the end of the twentieth century and the beginning of the twenty first when a "gold rush" in synthesis started with the publication of the original works of Fukuda (Fukuda and Utimoto, 1991), Teles (Teles et al., 1998), and Tanaka (Mizushima et al., 2002) on the homogeneous gold-catalyzed addition of water and alcohol to alkynes. The seminal contributions that sparked the interest in gold catalysis in the ending years of the twentieth century were due to Hashmi et al. who showed the catalytic reactivity of AuCl^3^ on cycloisomerization reactions of alkyne-based compounds leading to furans and arenes (Hashmi et al., 2000; Stephen et al., 2000). Since then the interest on this metal has increased notably as revealed by the vast number of publications on gold catalysis^1^. Located in group 11 of the periodic table, gold behaves as a soft carbophilic Lewis acid with the ability to stabilize an adjacent carbocation through back-donation. Relativistic effects in this atom promote the contraction of its *6s* orbital, which becomes the main reason for this relatively uncommon behavior across the periodic table (Gorin and Toste, 2007; Faza and López, 2015). Nevertheless, gold complexes are usually poorly-reactive in their precatalytic state and they need prior transformation *in-situ*, most commonly through the abstraction of one ligand, generally a chloride group. Among others, silver salts are the most used agents for this purpose (Ranieri et al., 2015). Furthermore, both the electronic nature of ligands (Wang et al., 2012; Ebule et al., 2016; Gung et al., 2016; Ferrer and Echavarren, 2018a) and of the counterion (Homs et al., 2014; Ciancaleoni et al., 2015; Jia and Bandini, 2015; Rocchigiani et al., 2015; Gatto et al., 2016; Yuan et al., 2018; Schießl et al., 2018a,b) have a significant influence on the reactivity of the gold-catalyzed processes.

**Figure 1:**
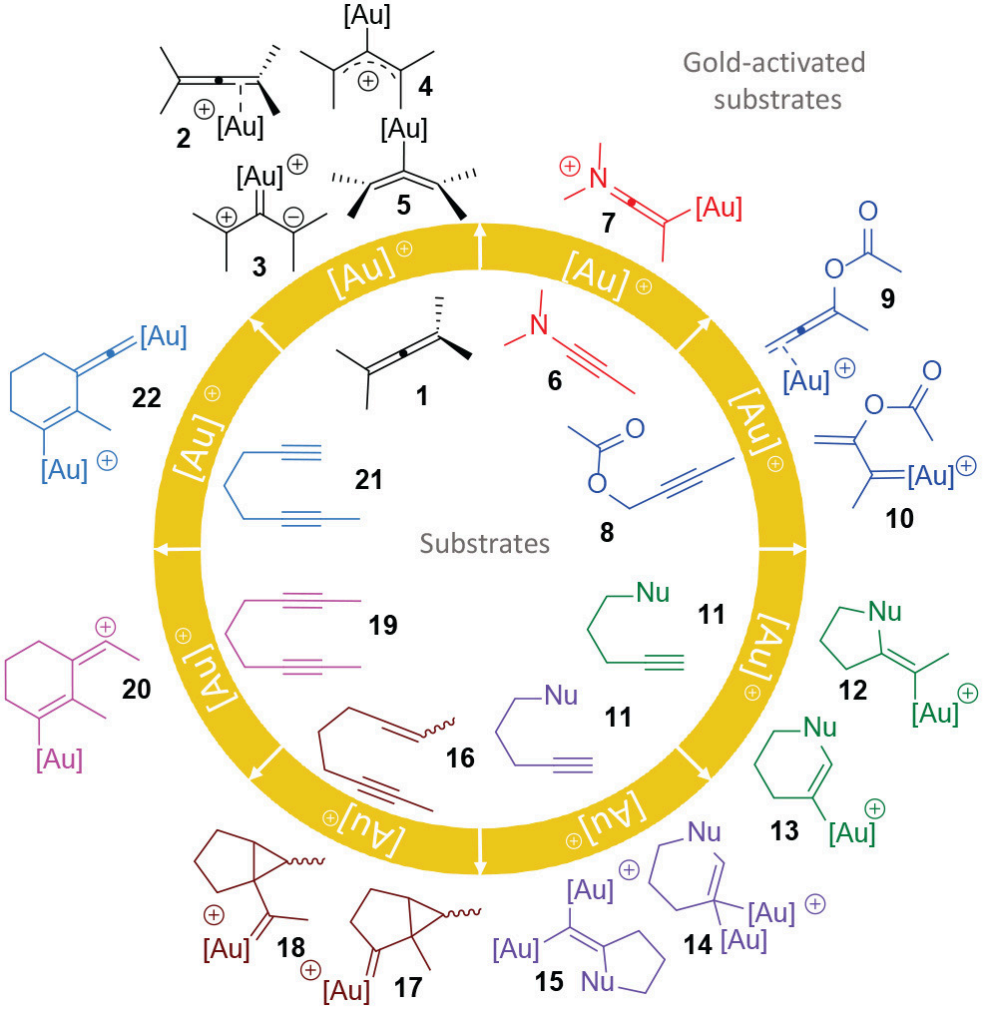
Gold activation reaction patterns toward different unsaturated substrates.

Thus, gold(I)- and gold(III)-complexes are mostly intended for the activation of unsaturated groups, such as allene and alkyne derivatives (Jones, 2015; Blons et al., 2018). Figure 1 shows the possible activation modes exerted by a generic cationic gold complex [Au]^+^ toward common starting materials which will be the subject of revision in this report. Four possibilities have been described for the coordination of gold to an allene moiety **1**: η^2^-coordinated complexes (**2**), zwitterionic carbenes (**3**), σ-allylic cations (**4**) or η^1^-coordinated bent allenes (**5**) (Soriano and Fernández, 2014). Cumulenic derivatives could also be formed under gold activation. The π-coordination of gold to the alkyne group of an ynamide substrate **6** promotes the formation of an electrophilic keteniminium ion **7** susceptible to a nucleophilic attack. Propargylic carbonate/ester substrates **8** are prone to rearrange under gold-catalysis, usually trough a 1,3- or 1,2-migration of the carbonate/ester group over the π-system toward either the allene intermediate **9** or the gold-carbene **10**, respectively (Ghosh et al., 2014; Swift and Gronert, 2016). Alkynes containing and internal nucleophilic functional group **11** can cyclize either in an *exo-dig* or *endo-dig* fashion leading to **12** and **13**. Otherwise, it is possible that two gold complexes coordinate simultaneously to the terminal alkyne **11** forming a σ,π-digold alkyne and then furnishing intermediates **14** and **15** (Cheong et al., 2008; Larsen et al., 2015). Cyclopropane gold carbene-like intermediates **17** and **18** are, respectively, achieved through an *endo-dig* and an *exo-dig* carbocyclization of the enyne **16** (Obradors and Echavarren, 2014). Two internal alkynes placed in an diyne **19** can react onto each other under gold-π-activation forming a conjugated vinyl cation intermediate **20**. Recently, the possibility of dual gold-activation has started to garner attention. It is a reactivity pattern for diyne systems, in which one gold center simultaneously enhances the nucleophilic character of the terminal alkyne trough a σ-bond while a second gold center turns the other alkyne motif more electrophilic trough a classical π-coordination (Cheong et al., 2008; Odabachian et al., 2009; Stephen et al., 2012; Ye et al., 2012; Hashmi, 2014). This reactivity is represented in the transformation of dyine **21** toward intermediate **22**. The diversity in the activation patterns shown by gold opens a large window of possibilities for the use of this metal as a powerful tool in the design and synthesis of relevant compounds in different fields. In fact, an increasing number of structurally complex molecules, such as natural products, biologically active compounds or polycyclic systems are reachable from synthetically accessible alkyne, alkene and allene reagents under metal-mediated isomerization process (Aubert et al., 2011; Yamamoto, 2012; Zhang et al., 2012; Stathakis et al., 2016; Hu et al., 2017; Herndon, 2018). In this sense, gold shines over other metals mostly due to its tolerance to diverse functional groups, low toxicity and the usually mild thermal conditions that are required to run these kind of transformations, in some occasions even showing tolerance to aqueous media or green solvents (Gatto et al., 2018).

This review focuses on homogeneous gold-mediated intramolecular (cyclo)isomerizations without the direct intervention of external agents in the intermediate mechanistic steps, except water in some unavoidable cases. It is organized according to both the kind of substrates to be activated by the gold complex: Allenes, 1,*n*-dienynes and 1,*n*-diynes and to the first mechanistic step in the global transformation such as carbocyclization, nucleophilic attack or carbonate/ester group rearrangement processes. Due to the overwhelming number of studies and the recently published reviews about gold-mediated isomerization reactions (Belmont and Parker, 2009; Fensterbank and Malacria, 2014; Yang and Hashmi, 2014; Dorel and Echavarren, 2015; Asiri and Hashmi, 2016; Day and Chan, 2016; Maes et al., 2016; Quintavalla and Bandini, 2016; Siva Kumari et al., 2016; Wei and Shi, 2016; García-Morales and Echavarren, 2018; Lee and Kumar, 2018; McGee et al., 2018; Toullec and Michelet, 2018), we will include here the most relevant contributions of the last years.

## 2. Gold-Catalyzed Isomerization Processes Involving Allenes

Allenes are cumulenic compounds in which one carbon atom stablishes a π-bond with two adjacent carbon centers, adopting an ideal bond angle of 180° (Patel and Bharatam, 2011; Soriano and Fernández, 2014). Both the structural architecture of this triatomic system and the electronic nature of the substituents on it direct the metal coordination modes and therefore the reactivity of the activated allene (Figure 1). In addition, the initial mode in which gold coordinates to the cumulenic motif, and the further transformations, determines the spatial disposition of the substituents observed in the isomeric products (Yang and Hashmi, 2014) and even turns the mechanistic pathway away from that of the uncatalyzed reaction (Mandal and Datta, 2018). Interestingly, gold complexes are able to catalyze the racemization of 1,3-disubstituted allenes, a process which has been theoretically studied for the particular case of IPrAuOTf acting as the catalyst (Li et al., 2016).

**Figure 2:**
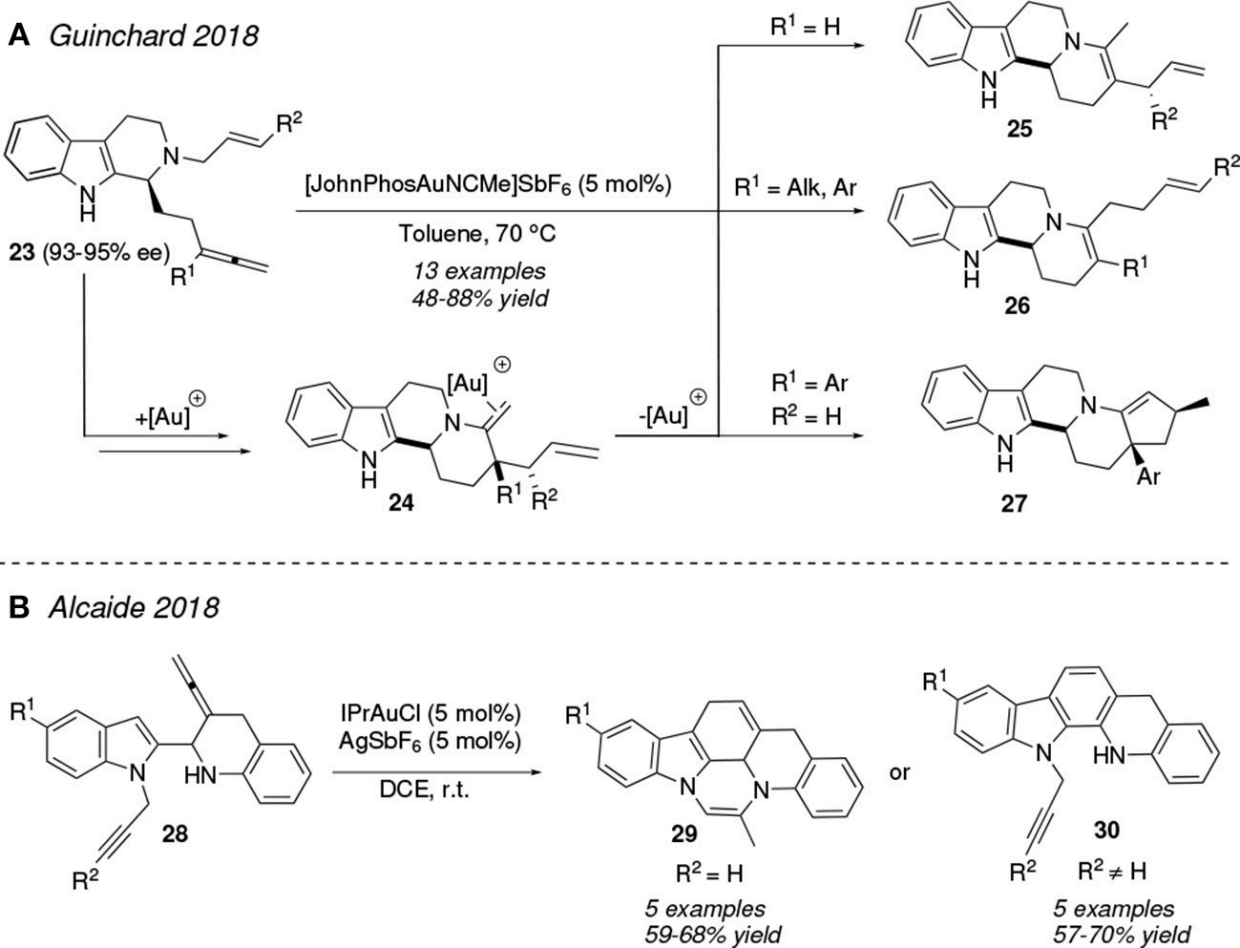
Gold-mediated cycloisomerization of allene derivatives.

In 2018, Guinchard et al. reported an elegant thermal ring closure of enantionenriched *N*-allyltryptamines **23** catalyzed by the Echavarren catalyst, [JohnPhosAuNCMe]SbF_6_ (Figure 2A). The initial gold(I)-activation of the allene motif promotes the tandem 6-*exo-dig*/aza-Cope rearrangement process affording intermediates **24**. The nature of the R^1^ and R^2^ substituents placed at both the original allene and alkene groups direct the subsequent evolution of **24** toward the different indolo[2,3-*a*]quinazolines **25**, **26** and **27**. Thus, for R^1^ = H **24** undergoes an isomerization process of the exocyclic C—C double bond yielding **25** whereas when R^1^ = alkyl, aryl the mechanism evolves trough a [3,3]-Cope rearrangement to afford compounds **26**. Pentacyclic substrates **27** were isolated for the special case of R^1^ = aryl and R^2^ = H (Gobé et al., 2018). In the same line, the (non-)substitution at the terminal position of the alkyne group contained in the initial indole-tethered amino allenynes **28** is the responsible for the divergent synthesis of the fused polyciclic indoles **29** and **30** (Figure 2B). [IPrAu]^+^ would activate the allene group toward a 6-*endo*-carboauration at the C3 atom of the indole ring as initial step whereas the subsequent hydroamination process over the *N*-pendant terminal alkyne ends in the hexacycle **29**. This process does not occur in the case of alkynes bearing bulky R^2^ groups. Pentacycles **30** are therefore accessed in these latter transformations (Alcaide et al., 2018).

**Figure 3:**
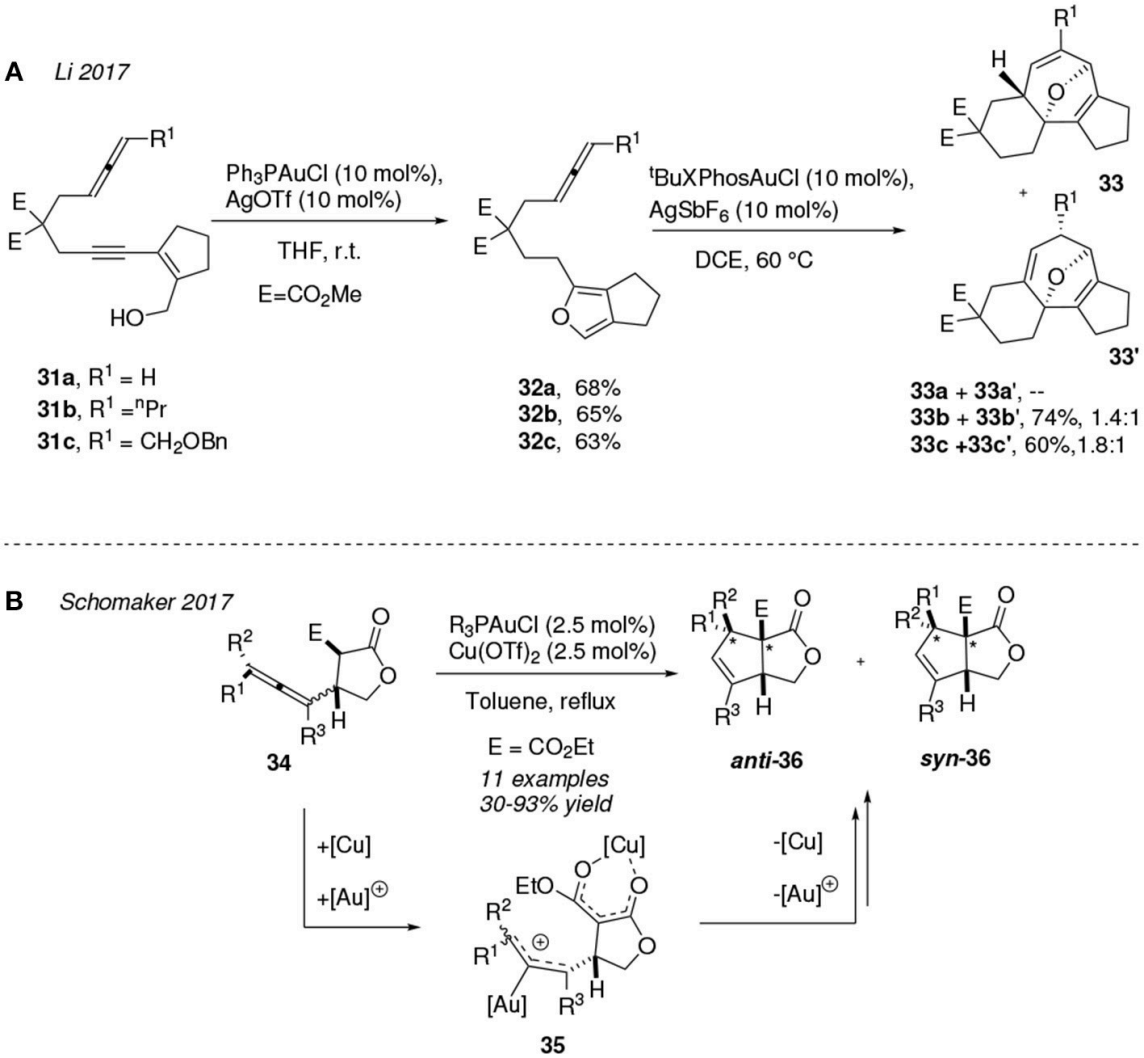
Gold-mediated cycloisomerization of allene derivatives.

Other gold-mediated carbocyclization reactions have been described for allenes. Daphnane/tigliane diterpene natural products contain a common scaffold based on a 5-7-6 carbotricyclic ring system. In this sense, Li et al. published a gold(I)-mediated sequential transformation of allenynes **31** into the polycyclic ethers **33** (Figure 3A). The authors noted that the overall direct gold-catalyzed process was unfruitful, requiring the optimization of a two-step sequence under different reaction conditions and gold(I)-complexes. Thus, [PPh_3_Au]^+^ promotes the initial *5-exo-dig* hydroalkoxylation onto the alkyne group toward intermediates **32** (Riedel et al., 2017) which subsequently undergo a furan-allene (4+3) cycloaddition under thermal treatment (60°C), and with the participation of [tBuXPhosAu]^+^, to afford the isomeric mixture of the desired product **33** plus **33'**. This last step is highly influenced by the nature of the R^1^ substituent at the allene site. Terminal allenes (R^1^ = H) are poor substrates for this (4+3) cycloaddition, producing a complex mixture of products whereas tricyclic systems **33b** and **33c** are obtained in good yields and with a moderate isomeric ratio for the target product **33** vs. the undesired ether (Li et al., 2017b).

In 2017, the combination of gold(I) and copper(II) complexes was reported as an efficient catalyst to promote the site- and regiocontrolled carbocyclization of a racemic mixture of allenes **34**. In order to explain the mechanistic sequence behind these transformations Schomaker et al. proposed a double metal activation in which Cu(OTf)_2_ would form a Cu-enolate chelate whereas [R_3_PAu]^+^ (R = Ph or Cy) would activate the allene motif (**35**) to then furnish the cylopentene species **36** in >1:1 dr (*anti:syn* of the two starred carbon centers). Remarkably, the stereochemistry at the junction of the two 5-member rings at **36** remains *syn* during the process. The different dr observed in products **36** respect to the 1:1 dr in the initial mixture of allenes **34** is reasoned assuming that the rate of the gold-activated allene epimerization is faster than that of the carbocyclization process at the intermediate **35** (Figure 3B) (Reeves et al., 2017).

**Figure 4:**
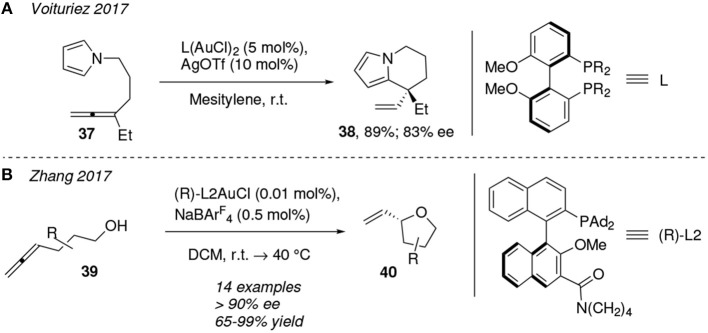
Cycloisomerization of allene derivatives by chiral gold-catalysts.

In the last few years many chemists have focused their efforts in developing enantioselective reactions, which is currently one of the most active arenas in the field of gold catalysis (Wang et al., 2014). An illustrative example is that published by Voituriez et al. in the course of the total synthesis of the natural product (-)-rhazinilam. They reported the use of a digold(I) complex attached to the chiral biphenyl-phosphine ligand L to mediate in the enantionselective 6-*exo-trig* cycloisomerization of the allene-functionalized pyrrole **37**, which leads to the tetrahydroindolizine derivative **38** in 89% yield and 83% ee. This reaction is conducted in a solution of mesitylene at room temperature (Figure 4A) (Magné et al., 2017).

In the same line, asymmetric gold-catalysis via the combined use of a non-spectator chiral ligand and a metal center has been described for the cyclization of the 4-allen-1-ol substrates **39** affording chiral 2-substituted tetrahydrofurans **40** (>90% ee, Figure 4B). The pendant amide group at the chiral binaphtyl-based ligand (R)-L2 seems to play a crucial role in the rate acceleration of the reaction due to its abilities to act as general base. Authors propose that the preferred gold-allene coordination is that in which that amide group is placed close to the alcohol group, whereby forming a hydrogen bond C=O⋯ H-O which increases the nucleophilicity of the oxygen atom and thus the rate of the reaction (Wang et al., 2017a).

Furthermore, the highly stereoselective cycloisomerization of optically active 4,5-alkadienoic acids leading to gamma-butyrolactones has been reported to occur under catalysis of chiral gold-complexes (Zhou et al., 2018a). Also, non-chiral gold catalysts have been reported to promote related cycloisomerizations of cumulenols (Alcaide et al., 2016) or allenols (Lempke et al., 2016). For this later example, the effects governing the mechanism of the gold-catalyzed attack mode of hydroxylamines onto allenes yielding either dihydrooxazine or *N*-hydroxypyrroline derivatives (that is O- vs. NH-attack and 5- vs. 6-member ring formation) were computationally studied and described in detail by Silva and coworkers in 2017 (Kiriakidi et al., 2017).

## 3. Gold-Catalyzed Isomerization Processes Involving an Initial Alkyne Activation

Most of the studies on gold-mediated isomerization reactions are devoted to the transformation of alkynes bearing a second insaturation (an alkene or alkyne group), that is 1,*n*-enyne and 1,*n*-diyne substrates (Jiménez-Núñez and Echavarren, 2008; Asiri and Hashmi, 2016; Day and Chan, 2016; Lee and Kumar, 2018). Nonetheless, cycloisomerization processes have also been described in molecules containing solely a reactive alkyne or alkene group. This is the case of the gold(I)-directed ring-contraction process of cyclooctyne to 5-member bicyclic alkenes (Das et al., 2016) or the intramolecular hydroamination of 6-alkenyl-2-pyridones to yield 1,6-carboannulated 2-pyridones (Timmerman et al., 2017). In those circumstances where both types of unsaturations are present, the well-known preference of gold for alkyne over alkene groups is rationalized in terms of the HOMO-LUMO energy gap of the coordinated π-systems rather than through intrinsic considerations of the metal itself (Gorin and Toste, 2007). Henceforth, (cyclo)isomerization processes initiated by the gold activation of an alkyne group will be described.

### 3.1. Gold-Catalyzed Cycloisomerization of Ynamides

**Figure 5:**
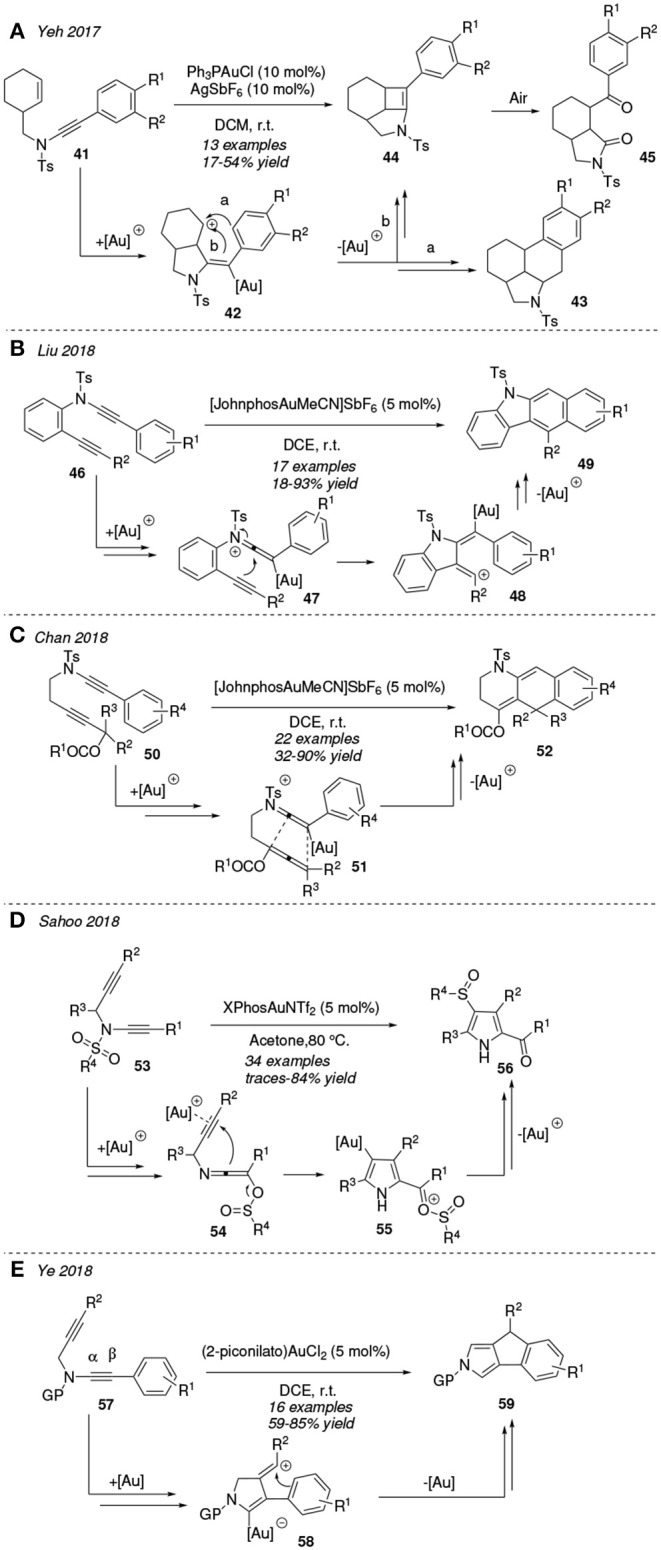
Gold-mediated cycloisomerization processes of ynamides.

Ynamides are special amines substituted by an alkyne group and an electro-withdrawing group which modulate its stability and reactivity (Pan et al., 2016). Yeh et al. reported the gold(I)-catalyzed double cyclization of the 3-enynamides **41** in dichloromethane at room temperature (Figure 5A). The authors propose an initial cyclization involving the gold-activated alkyne motif and the pendant endocyclic C=C bond that provides vinyl cation **42**. This cationic intermediate **42** could cyclize forming either a 6-member ring, by attack of the pendant phenyl ring onto the deficient carbon center (path *a* toward **43**, traces) or a 4-member ring, involving the exocyclic alkene (path *b* leading to **44**). The strained cyclobutene compounds **44** are quickly oxidized when exposed to air, affording the respective 1,4-diketones compounds **45**, which are isolated as major products (Zhong et al., 2017).

In 2018, Liu et al. designed a new route toward benzo[*b*]carbazoles **49** involving the [JohnPhosAuNCMe]^+^-catalyzed cycloisomerization of the ynamide-ynes **46** via a formal dehydro-Diels-Alder reaction at room temperature (Figure 5B). Initial gold-activated keteniminium ions **47** undergo a cyclization affording the vinyl cations **48** which evolve toward the final tetracycles **49** by a benzoannulation reaction (Xu et al., 2018). The same catalyst, [JohnPhosAuNCMe]SbF^6^, mediates the transformation of ynamides **50** into tetrahydro[*g*]quinolines **52** (Figure 5C). An initial [3,3]-sigmatropic rearrangement of the acetate group and activation of the ynamide group would yield the keteniminium ion **51** which would experiment a formal [4+2]-cycloaddition yielding **52**. This protocol is efficient even in the presence of air and moisture, indicating the tolerance of the gold catalysts to diverse reaction conditions (Chen et al., 2018).

A very interesting case is that of *N*-substituted ynamides decorated with functional groups with migratory abilities. Figure 5D shows the unconventional thermal transformation of *N*-sulfonyl ynamides **53** to 4-sulfinylated pyrrol derivatives **56** in presence of XPhosAuNTf_2_ catalyst. The authors proposed a mechanism initiated by a [1,3]-sulfonyl migration from the N atom to the distal carbon atom of the alkyne fragment resulting in intermediate **54**. The subsequent gold-activation of the alkyne moiety promotes an umpolung at the unsaturations and facilitates a 5-*endo*-dig cyclization toward **55**, which is transformed into the indole **56** via a deaurative [1,5]-sulfinylation process. This kind of sulfonyl/sulfinyl shift proceeds in a regioselective fashion. These results were also supported by a computational study (Sahoo et al., 2018).

Furthermore, gold(III)-complexes are able to promote the activation of ynamides toward cycloisomerization reactions. In 2018, Ye et al. reported the practical synthesis of indeno[1,2-*c*]pyrroles **59** in good yields under mild conditions from *N*-propargyl ynamides **57** and with gold(III) catalysis. In contrast to the usual metal-catalyzed cyclization of π-tethered ynamides over the central α-carbon atom, authors reported a regioselective attack on the β-carbon of the ynamide **57** leading to the vinyl cation **58**, mostly associated to a lower ring strain of the formed pentacycle with respect to the four-member ring alternative (Figure 5E) (Shen et al., 2018).

### 3.2. Gold-Catalyzed Cycloisomerization of 1,*n*-dienynes and 1,*n*-diynes Containing a Propargyl Carbonate/Ester

**Figure 6:**
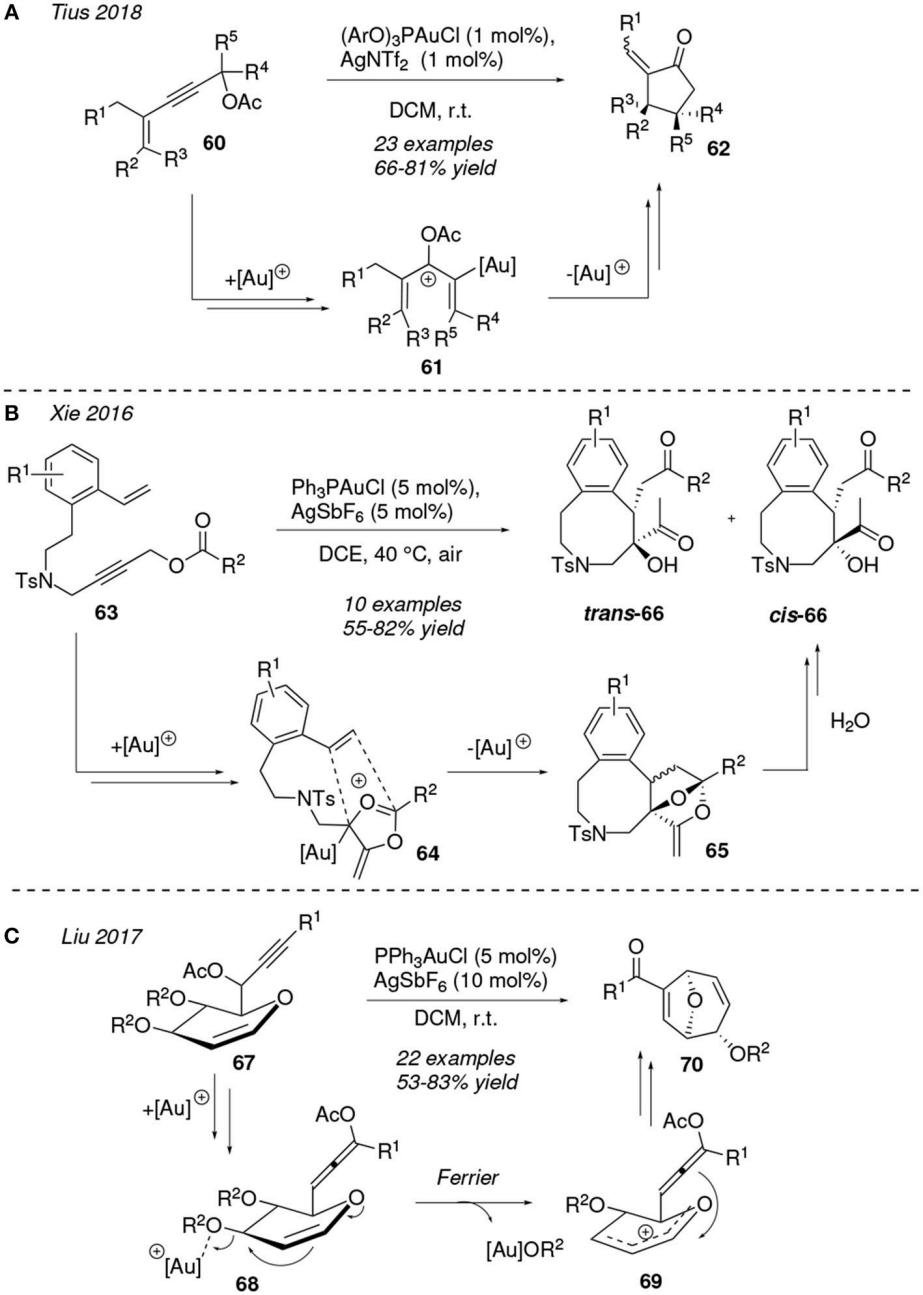
Gold-mediated cycloisomerization of 1,*n*-dieniynes started by an acyloxy shift process.

In the last 3 years a great number of studies devoted to gold-mediated cyclizations of compounds containing a propargylic carbonate/ester motif have been reported. It is worth to note that the usual initial carboxy rearrangement determines the fate and further transformations that encompass this chemistry. For this reason substrate design is key to successfully obtain the desired target molecule through these reactions. Recently, Zhang et al. introduced the application of frustrated Lewis pairs as a synthetic strategy, that is, basic tertiary amine as ligands to design gold cationic complexes which improve the regio- and stereoselective ratios of propargylic ester isomerizations (Wang et al., 2017b). In this sense, related 1,*n*-enyne derivatives are well-exploited reactants and powerful synthethic tools to build carbonyl-based compounds. Figure 6A describes the diasterospecific synthesis of cyclopentanones **62** owning two contiguous stereogenic carbons published by Tius et al. The authors use a gold(I) complex to catalyze a tandem [1,3]-OAc shift/cyclization/acetate hydrolysis process. This cycloisomerization reaction can be conducted at room temperature with substrates **60** bearing different functional groups such as esters, -CF_3_ or alkyl chains. The high diastereoselectivity observed in the transformation is related to the rapid alkenyl isomerization of the pentadienyl intermediate **61** (Congmon and Tius, 2018).

A couple of years earlier, the preparation of benzazocines **66** was accomplished from terminal 1,9-enynyl esters **63** through a gold-catalyzed cascade reaction. Xie et al proposed the initialization of the mechanism with a 1,2-acyloxy shift that further evolves with a *O*-cyclization to yield 1,3-dipolar intermediates **64**. These **64** species engage in a [3+2] cycloaddition reaction involving the terminal alkene to afford the polycyclic scaffolds **65**, which convert into the final substituted benzocines **66** by a hydrolisis process. Two different diastereoisomers were isolated and fully characterized, the *trans* isomers **66** being the major products of the reaction (Figure 6B) (Feng et al., 2016). The same group has reported on the successful attempts to obtain complex tetracyclic frameworks by appliying a similar gold-catalyzed protocol on related linear enynyl esters (Sun et al., 2017).

Liu et al. published the gold(I)-mediated diastereoselective formation of 8-oxabicyclo[3.2.1]octanes **70** from glycal bearing porpargylic esters **67** (Figure 6C). The authors proposed that the [PPh_3_Au]^+^ catalyst has a dual role during the course of the reaction. First, the gold(I)-complex activates the alkyne group to facilitate a 1,3-acyloxy migration leading to the allene intermediates **68**. Further along into the mechanistic pathway, the metal acts as Lewis acid promoting the intramolecular Ferrier reaction with the departure of the alcoxy group, which is integrated as a new ligand in the gold complex [Au]OR^2^ furnishing the oxocarbenium species **69**. Lastly the bicyclic structure of **70** is formed by cyclization and formation of AcOR^2^ as a by-product (Liao et al., 2017).

**Figure 7:**
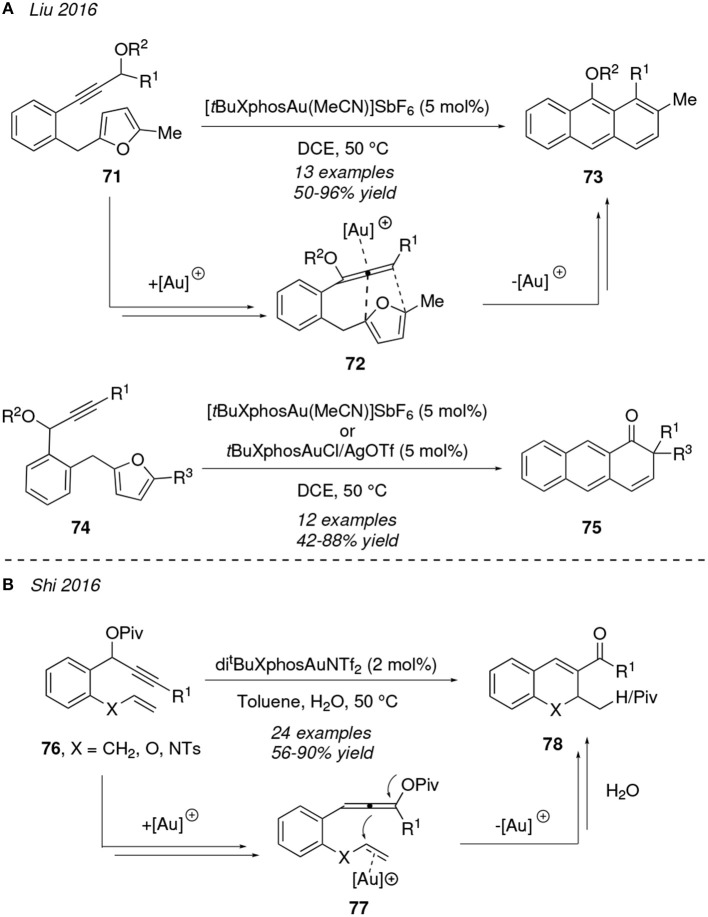
Gold-mediated cycloisomerization of 1,*n*-dienynes started by an acyloxy shift process.

In conjunction with the latter works, functionalized anthracenes **73** can also be generated under thermal conditions (50°C) with bulky gold(I)-catalysts promoting the cyclization of 2-(2-ethynylbenzyl)furan featuring propargyl carbonate or ester groups **71**. Intermediates **72** are proposed to be formed after a 3,3-rearrangement of the propargyl carboxylate OR^2^ moiety, and then evolve to products **73** via a [4+2]-cycloaddition between the furanyl system and the distal C=C double bond of the allene. A similar protocol was applied in the cycloisoimerization of the related 1,5-furan-ynes **74** to obtain antracen-1(2*H*)ones **75**; in this case a late 1,2-R^1^ shift is required after the [4+2]-cycloaddition step to afford the final ketone (Figure 7A) (Sun et al., 2016).

An unusual chemoselective gold-activation of alkenes over allenes using sterically bulky ligands as di^*t*^BuXphos has been described by Shi et al. Substituted bicyclic[4.4.0]dihydronaphthalenes **78** were accessed from the dienynes **76** through an initial 1,3-OPiv rearrangement and subsequent alkene-activation at intermediate **77**, which then experiments an allene-ene cyclization and hydrolysis. Interestingly, the pivaloyl group (Piv) migrates selectively during the formation of hydrobenzopyranes (**78**, X = O) as indicated in Figure 7B (Thummanapelli et al., 2016).

**Figure 8:**
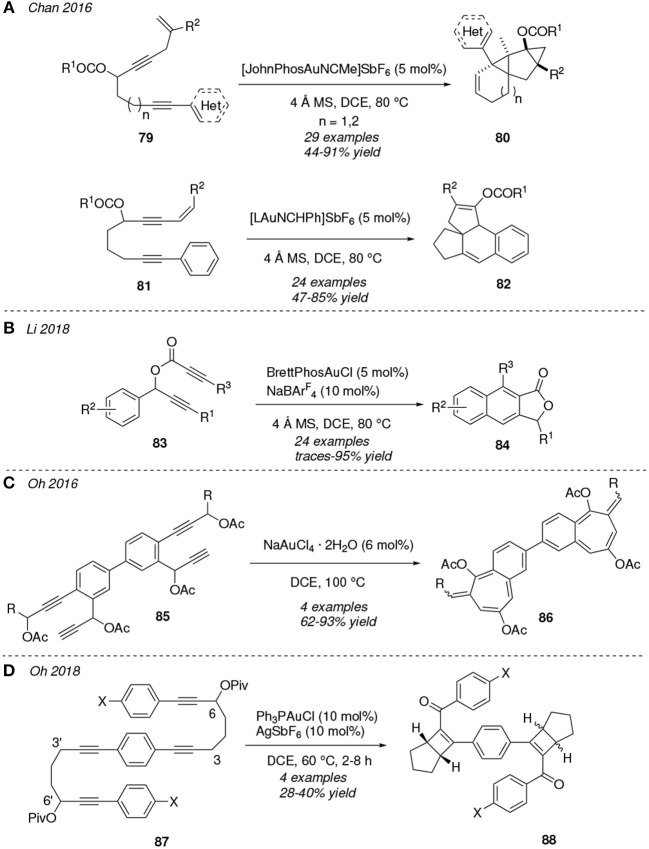
Gold-mediated cycloisomerization of 1,*n*-diynes started by an acyloxy shift process.

As in the case of 1,*n*-enynes, reactions initiated by migration of a carbonate or ester group have been subject of study in related 1,*n*-diyne derivatives (Day and Chan, 2016; Li et al., 2017a). Chan et al. reported in two different studies the gold(I)-mediated cycloisomerization of 1-en-4,*n*-enynes **79** (Chen et al., 2016a) and 1-en-3,9-enynes **81** (Figure 8A) (Rao et al., 2016). Both reactions were conducted under the same solvent and temperature conditions (DCE, 80°C) but with different gold catalysts. Thus, for the conversion **79**→**80** [JohnPhosAuNCMe]^+^ was the active cationic gold species which provided the best results, whereas in the case of the cycloisomerization of the diyne **81** [LAuCHPh]^+^ was employed. In the latter, therefore a cationic catalyst featuring a *N*-heterocyclic carbene ligand is used. In both cases the mechanistic route would be initiated by the gold-activation of the propargyl moiety promoting a 1,3-acyloxy transfer followed by several cyclizations. In fact, the relative position of the olefine moiety determines whether the evolution of the mechanisms at the first cyclization occurs through either a 1,4-eneallene cycloisometization toward **80** or a metallo-Nazarov cyclization toward the tetracycles **82**. In 2018, the synthesis of naphtho[2,3-*c*]furan-1(3-*H*)-ones **84** was reported via cycloisomerization of propargylic ynoates **83**, under thermal conditions with BrettPhosAuCl as catalyst and in presence of ${\text{NaBAr}}_{4}^{F}$ as an activator of the gold complex (Figure 8B) (Li et al., 2018a). The reaction mechanism would involve a [3,3]-rearrangement of the propargyl ester leading to a carboxyallene intermediate followed by an intramolecular Diels-Alder cyclization. The authors were able to synthesize up to 24 examples of **84** combining diverse alkyl and aryl substituents and they also describe a new method for the *in-situ* generation of carboxyallene intermediates.

An interesting formation of primitive dendrimer systems containing bicyclic structures from substrates incorporating two sets of dialkynes were described by Oh et al. The hydrated NaAuCl_4_ mediates in the thermal cycloisomerization of **85** trough a dual 1,3- and 1,2-acyloxy transposition at the terminal and inner alkyne moieties, respectively, followed by an annulation process leading to the benzo-fused 7-member cycles **86** (Figure 8C) (Lee et al., 2016). In a related work, the [Ph_3_PAu]^+^-activation of propargyl ester motifs promotes the 1,3-acyl shift/[2+2]/hydrolysis cascade reaction of the diynes **87** furnishing a diastereomeric mixture of the polycyclic systems **88** (Figure 8D). The authors were able to resolve the diasteromeric mixture and even extend this protocol to other diynes in which the pivaloate substituents are located at positions 3 and 3' (Lee et al., 2018).

### 3.3. Gold-Catalyzed Cycloisomerization Processes of 1,*n*-enynes and 1,*n*-diynes Initiated by a Nucleophilic Attack Onto the Alkyne Group

**Figure 9:**
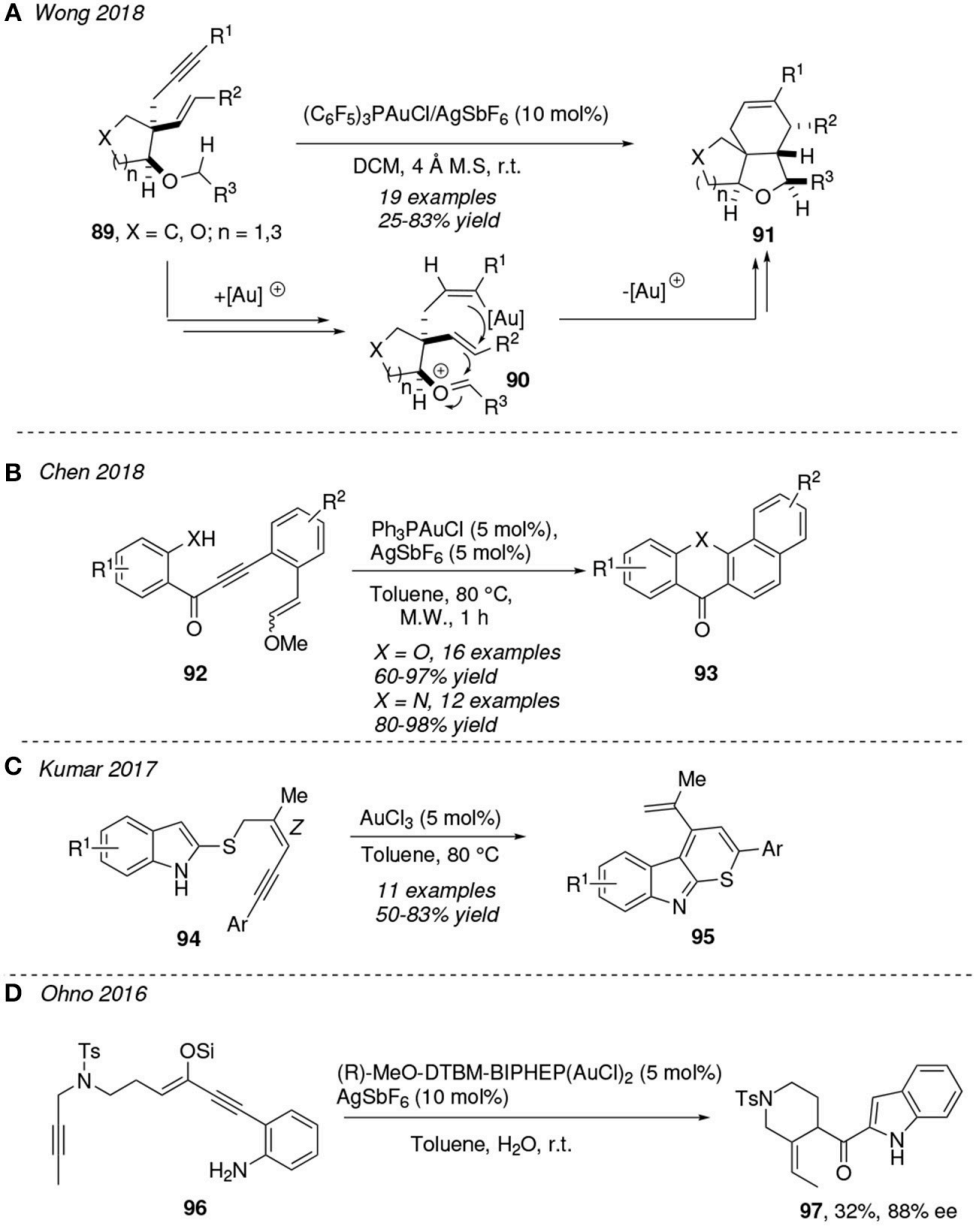
Gold-mediated cycloisomerization reactions of 1,*n*-enynes containing an internal atom-centered nucleophile.

Gold is able to activate an alkyne group toward a nucleophilic addition and, for instance, make it susceptible to act as the receptor motif in a hydride shift reaction (Xie et al., 2014; Nahide et al., 2018). In this sense, Wong et al. reported the gold-alkyne activation of 1,5-enynes **81** toward a rare 1,6-hydride shift leading to the gold-activated oxonium intermediate **90**, which then experiments a Prins-type cascade forming two new C—C bonds and furnishing the final tricyclic system **91** (Figure 9A) (Lu et al., 2018). This mechanistic proposal is supported by deuterium-labeling cross-over experiments. According to the authors, there is no precedent of cycloisomerizations initiated by a gold-catalyzed 1,6-hydride transfer reaction. This protocol provides rapid access to fused polycyclic compounds presented in many bioactive natural products as Nominine or Walsuranin B.

An activated alkyne group could act as electrophile in an intramolecular nucleophilic attack if a functional group present within the system has such nature. An experimental gold(I)-mediated double 6-*endo* cascade cyclization strategy of enynes **92** to form benzoxanthenone and benzoacridone derivatives **93** has been described by Cheng et al. (Figure 9B). The tandem process starts with an X-attack (X = O, N) to the alkyne moiety and further carbocyclization involving the pendant alkene motif, ending with the formation of both C—N and C—C bonds and releasing a methanol molecule. This reaction is conducted in toluene at 80°C under microwave irradiation; this provides a better yield when compared with conventional thermal treatment (Xiong et al., 2018). Before this study, the same group had reported a similar protocol to obtain benzo[*a*]carbazoles via gold(I)-mediated cycloisomerization of structurally related *ortho*-alkynylanilines in toluene at 100°C (Peng et al., 2017). Likewise, gold(III)-complexes have been reported to be excellent catalysts in similar transformations. Kumar et al. developed an elegant gold(III)-mediated strategy for the synthesis of substituted thiopyrano[2,3-*b*]indoles **95**. This implies that enyne tethered indole sulfides **94** undergo a tandem σ-bond migration/*6-endo-trig* cyclization/oxidative aromatization process. The authors highlight the significance of the *Z* orientation of the C=C bond to assist the rearrangement step (Figure 9C) (Jha et al., 2017).

Besides, the use of bimetallic complexes has become more popular in gold chemistry during the last years (El Sayed Moussa et al., 2016; Trommenschlager et al., 2017; Arif et al., 2018). The synthesis of the (+)-conolidine alkaloid has been reported via a gold(I)-catalyzed cascade cyclization of the conjugated enyne **96**. The bulky bimetalic gold complex (R)-MeO-DTBM-BIPHEP(AuCl)_2_ bearing a chiral ligand is used as effective precatalyst to form the ketone intermediate **97** (32% yield and 88% ee) (Figure 9D) (Naoe et al., 2016). Recently, Zi et al. achieved the first desymmetrization of prochiral bisphenols via gold(I)-catalyzed enantioselective hydroetherification of alkynes. This protocol was applied successfully in (di)alkyne compounds bearing P-stereogenic centers using the bimetallic chiral precatalyst (S)-DTBM-Segphos(AuCl)_2_ (Zheng et al., 2018).

**Figure 10:**
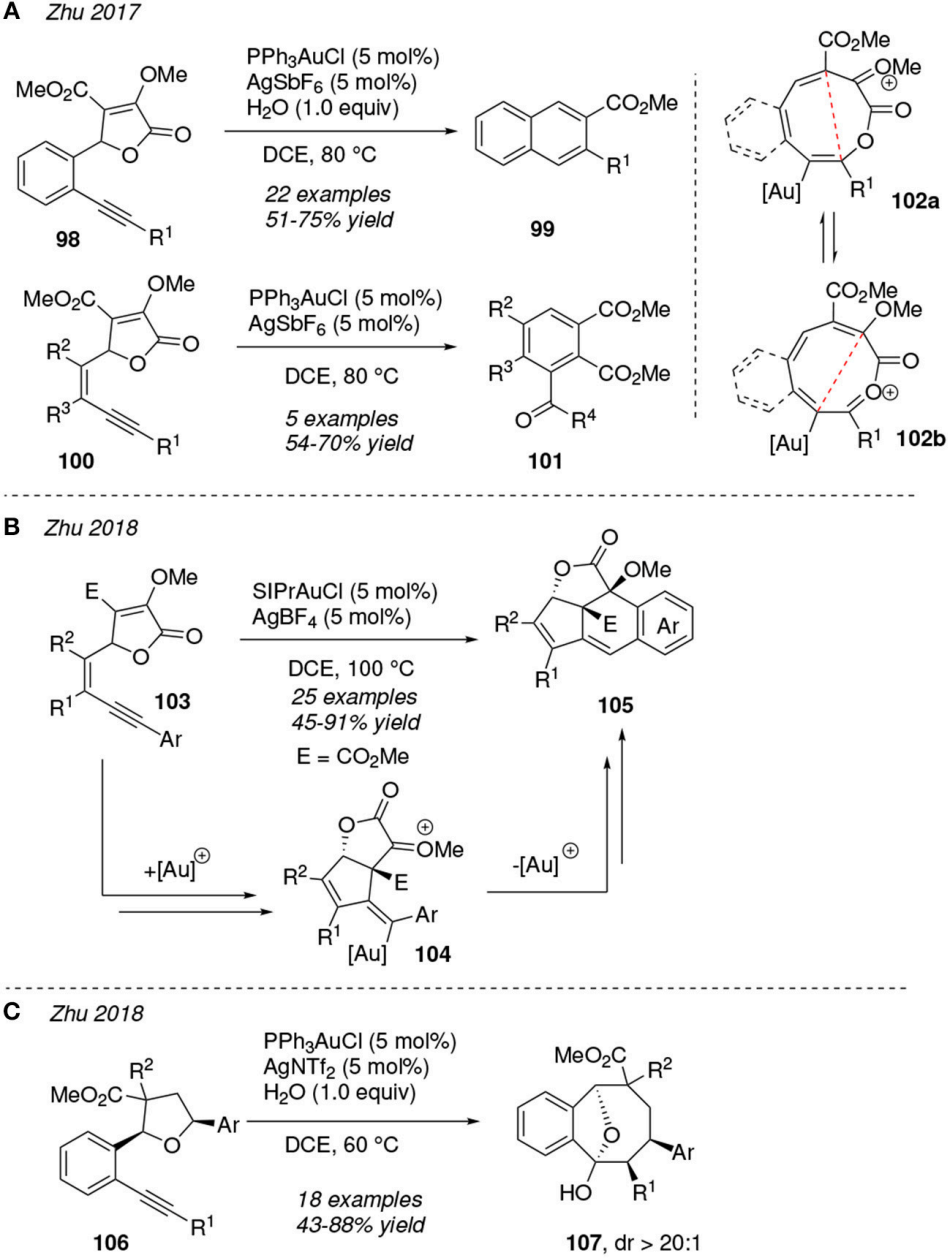
Gold-mediated cycloisomerization of enyne-lactones.

Several reports pivoting about cycloisomerization reactions of enyne-lactones by gold(I)-catalysis have been published by Zhu et al. (Figure 10). Under [Ph_3_PAu]^+^ catalysis, the lactone-enynes **98** and **100** are proposed to experiment a ring-expansion reaction of the lactone ring affording the previously unknown 2-oxoninium intermediate **102a** which exists in equilibrium with **102b**. The presence of the benzo-fused ring at intermediates **102** displaces the equilibrium reaction toward the most stable 2-oxoninium **102a**, whereas, in its absence, intermediate **102b** is the preferred product. Both intermediates **102a** and **102b** then undergo an interesting 6π electrocyclization reaction and subsequent aromatization process toward compounds **99** and **101**, respectively. The addition of one equivalent of water is required for the formation of **99** whereas the diester **101** is isolated even in anhydrous media (Figure 10A) (Luo et al., 2017). On the other hand, the nature of the ligand at the gold(I)-catalyst seems to have a high influence in the evolution of the mechanism allowing the transformation of related enyne-lactones **103**. Thus, benzo-fused polycyclic compounds **105** are achieved using a carbene derivate gold complex as precatalyst (SIPrAuCl) in presence of the activating partner AgBF_4_ (Figure 10B). The authors reasoned that an initial vinyl ether addition onto the activated-alkyne toward the **104** motif might be favored rather than an oxygen attack, as in the previous transformations, mostly due to the strong σ-donor and weak π-acceptor nature of the SIPr ligated gold center (Luo et al., 2018). If the lactone motif is replaced by a tetrahydrofuran group as in compound **106**, then the bicyclo **107** is obtained via a gold(I)-catalyzed *5-exo-dig*/[1,3] O-to-C tandem process in a diastereoselective fashion (Figure 10C) (Zhang et al., 2018a). With this set of papers Zhu et al. demonstrated the versatility of diverse gold(I) complexes in the ring-expansion process of lactones and related structural motifs via a nucleophilic attack onto an activated alkyne group.

**Figure 11:**
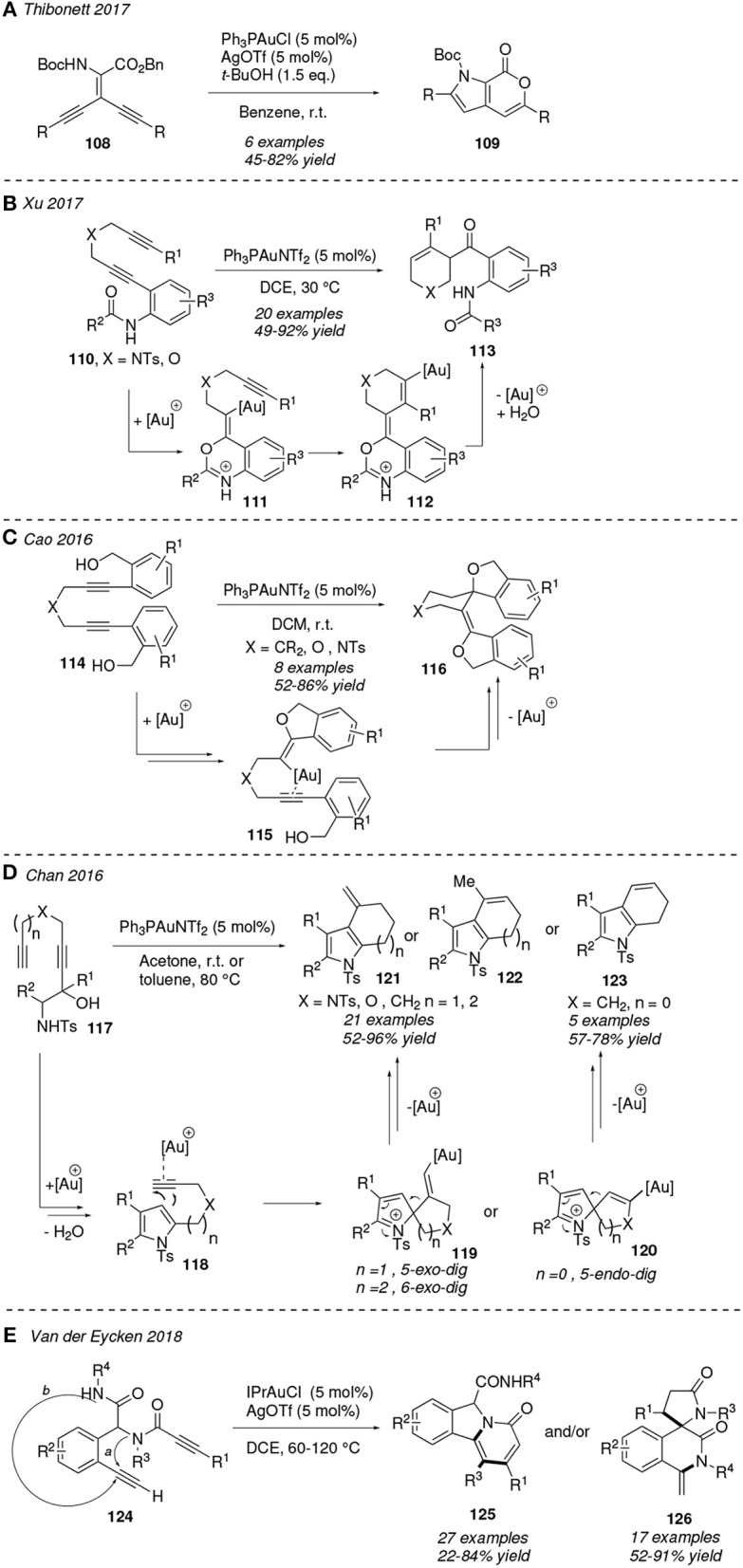
Gold-mediated cycloisomerization of 1,*n*-diynes containing an internal atom-centered nucleophile.

1,*n*-Diynes are prone to be transformed into valuable heterocyclic systems through the attack of an internal nucleophile, in particular -NH, -CO, or -OH groups, when present, either onto one or both of the gold π-activated C—C triple bonds. Lamellarins are a group of pyrrole alkaloids based in a pyrano[3,4-*b*]pyrrol-7(1*H*)-one scaffold which present anticancer activity. Thibonett et al. designed a gold(I)-catalyzed cycloisomerization of the 1,4-diynes **108** furnishing the substituted pyrano-pyrrol-ones **109** via two consecutive intramolecular *5-endo-dig* and *6-endo-dig* additions to both activated alkyne motifs (Figure 11A). The former cyclization proceeds involving the amine group in the formation of the pyrrol ring, whereas in the latter it is one of the ester groups who is involved in the formation of the pyranone ring (Delaye et al., 2017).

In 2017, Xu et al. reported an elegant gold(I)-catalyzed cyclization/carbonylation cascade reaction of diynes containing an amide group which operates under mild reaction conditions. Thus, 1,6-diynes **110** undergo a *6-exo-dig* cyclization with the carbonyl oxygen of the amide group leading to the 1,3-oxazine gold intermediate **111**, which is then intercepted by the remaining alkyne affording the intermediate **112**. After protodeauration and hydration β,γ-unsaturated ketones **113** are obtained in good yields (Figure 11B) (Bao et al., 2017). Otherwise, 1,6-diynes bearing *ortho*-benzyl alcohols **114** evolve to dihydroisobenzofuran derivatives **116** trough a gold(I)-catalyzed protocol initiated by two consecutive unsual *5-exo-dig* hydroxyalkilation processes and a further Prins-like cyclization. Authors reasoned the formation of the *5-exo-dig* cyclized product taking into account the more favorable six-membered ring chelated gold complex **115** when compared to the alternative bigger chelate, which would result in a more usual *6-endo-O*-cyclization (Figure 11C) (Hashmi et al., 2007). This kind of transformation has not only been described as regioselective but also as stereoselective at the spiro-quaternary center placing the oxygen atom in the axial disposition (Cao et al., 2016).

Chan et al. designed an efficient method to prepare azacycle-fused pyrroles **121-123** by gold(I)-catalyzed dehydrative cycloisomerization of β-amino-1,*n*-diynols **117**. The mechanistic sequence would be initiated with the formation of the pyrrole ring (**118**) promoted by the nucleophilic attack of the pendant amine toward the closer gold-activated alkyne and departure of a water molecule. A subsequent gold activation of the remaining alkyne motif would promote a cyclization leading to the intermediates **119** and **120**. The type of cyclization depends on both the length of the alkyl chain and the nature of the X linker (X = -NTs, O or CH_2_), as is shown in Figure 11D. Furthermore, the intermediate **119** is the precursor of the fused pyrroles **121** and **122** whereas pyrroles **123** result from the expansion and further protodeauration of **120** (Kothandaraman et al., 2016). The nature of the substituents of the amide groups at the diynes **124** direct the gold(I)-catalyzed formation of the lactams **125** and **126**. Thus, for a bulky R^4^ group and an electron-rich migrating R^3^ group path *a* is more favorable and involves a tandem *N*R^3^-nucleophilic cyclization/1,3-R^3^ migration/1,5-enyne cycloisomerization process to yield **125**. In contrast, if R^3^ is a poor shifting group, the dienynes **124** evolve trough a cascade hydroamination/Michael addition reaction (path *b*) to afford the spirocycle **126** (Figure 11E) (Li et al., 2018b).

### 3.4. Gold-Catalyzed Carbocyclization Processes of 1,*n*-enynes and 1,*n*-diynes

**Figure 12:**
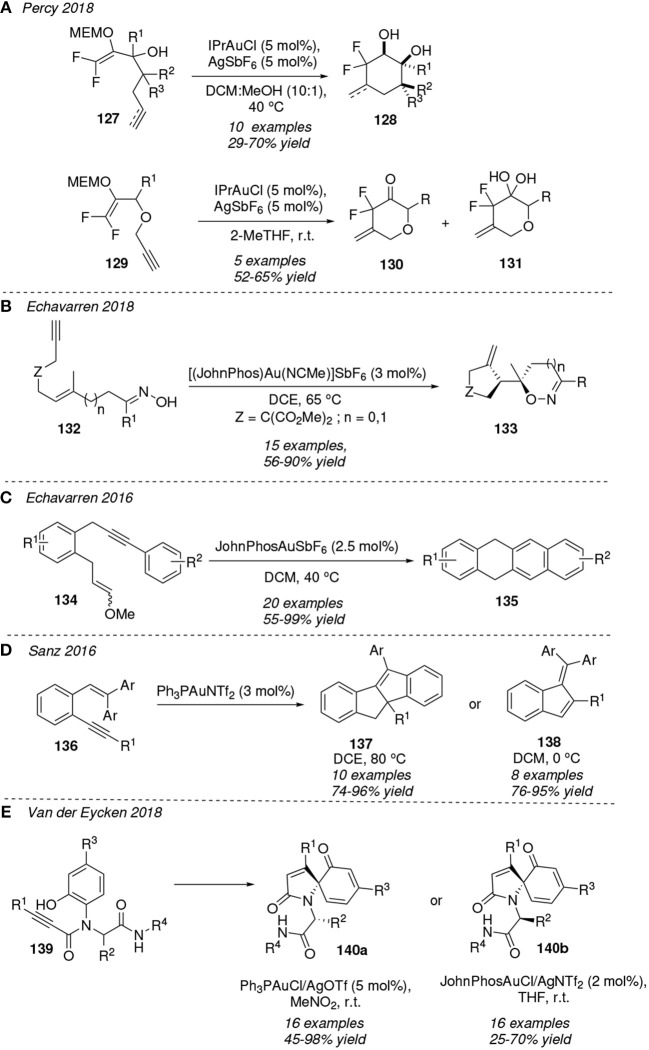
Gold-mediated cycloisomerization of 1,*n*-enynes.

Those processes in which 1,*n*-enyne substrates are transformed into isomeric products trough an initial carbocyclization step are very well documented. In 2018, Percy et al. have shown for the first time the intramolecular carbocyclization of difluorinated enol acetals bearing a pendant unsaturated group **127** leading to the cyclohexanone scaffolds **128** in a moderate diastereoisomeric ratio (Figure 12A). The reaction is conducted in a mixture of dichloromethane and methanol solvents at 40 °C in presence of IPrAuCl as precatalyst and AgSbF_6_ acting as activator agent of the previous one. The carbonyl group is further reduced to obtain difluorinated diols. Besides, a mixture of difluorinated pyran scaffolds **130** and **131** are isolated, the gem-diol derivate **131** being the major product when using propargyl ethers **129** as starting reactants and 2-MeTHF as solvent (McCarter et al., 2018).

In a very recent publication, Echavarren et al. reported the gold(I)-catalyzed cascade cyclization reaction of oxime-tethered 1,6-enynes **132** furnishing the substituted oxazines (*n* = 1) and dihydroisoxazoles (*n* = 0) **133** in a diastereospecific fashion and very good yields (Figure 12B). The mechanism ruling this transformation has been proposed to proceed stepwise through an intramolecular addition of the O atom of the oxime onto the transient gold-carbene intermediate, as revealed by the DFT computations performed. The *Z* orientation at the C=N bond of the oxime group is determining to obtain **133**, otherwise, with the *E*-oxime variant, dihydropyrrole *N*-oxides are isolated. The latter substrates resulted from the initial *N*-attack of the oxime over the gold-carbene intermediate (Muratore et al., 2018).

The use of gold-homogeneus catalysis to achieve complex poly-annulated-cyclic systems is also gaining attention as, for instance, in the formation of the biologically active compound (+)-aureol (Wildermuth et al., 2016). In this sense, stable functionalized hydroacenes **135** are easily accessible up to nine rings trough a gold(I)-catalyzed cyclization of aryl-tethered 1,7-enynes **134**, under mild reaction conditions (Figure 12C). A great number of different substituents R^1^ and R^2^ decorating the aryl motifs or even fused aromatic rings are compatible with the conditions, revealing the high synthethic value of this protocol (Dorel et al., 2016).

In 2016, Sanz et al. demonstrated that β,β-diaryl-*o*-(alkynyl)-styrenes **136** are transformed at 80 °C into dihydroindeno[2,1-*a*]indenes **137** under gold(I)-catalysis whereas benzofulvenes **138** are obtained at 0 °C . The formation of the tetracycles **137** implies a formal [4+1] cycloaddition trough a tandem 5-*endo*-cyclization-diene/iso-Nazarov cyclization process (Figure 12D) (Sanjuán et al., 2016). A computational study for related gold(I)-mediated transformations in which the initial styrenes bear alkyl groups at the β positions has been reported by Zhou et al. (2018b) The authors concluded that the reaction would evolve trough a [1,2]-H shift on the isopropyl moiety rather than a cyclopropane expansion, as suggested in the experimental work (Sanjuán et al., 2015).

The Van der Eycken research group has reported the elegant one-pot synthesis of spirocyclic pyrrol-2-one-dienones **140** via a gold(I)-catalyzed intramolecular Friedel-Craft reaction of the Ugi adducts **139** (Figure 12E). Two diastereomeric dienones were isolated depending on both the gold-catalyst and the reaction conditions. Taking the **140** yields into account and supported by a conformational theoretical analysis, the authors concluded that the isomer **140a** resulting from the catalytic treatment with Ph_3_PAuCl is energetically more favorable than that derived from the bulkier JhonPhosAuCl precatalyst (**140b**) (Nechaev et al., 2018). A related work had been previously published by the same group (He et al., 2017).

**Figure 13:**
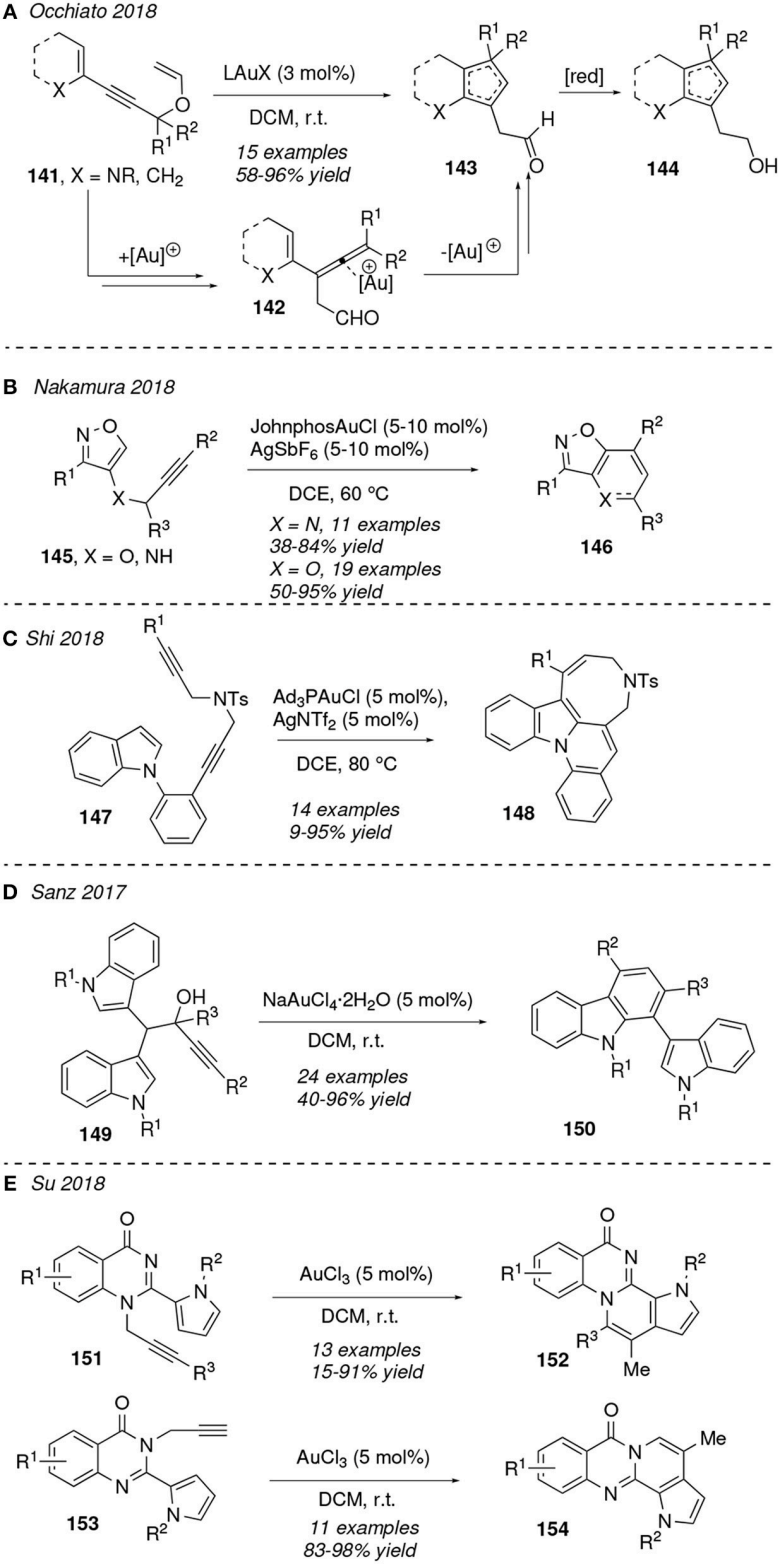
Gold-mediated cycloisomerization of 1,*n*-enynes.

Continuing with carbocyclization processes, it was reported that several gold(I) salts (L = t-Bu_3_P, IPr; X = [OTf]-, [NTf_2_]^−^, [SbF_6_]^−^) activate the 1,5-enynes **141** toward gold-allenes **142** via a [3,3]-rearrangement. The subsequent tandem Nazarov cyclization/[1,2]-H shift process allows the formation of the fused cyclopentadienes **143**, which were exposed to a further reduction to obtain primary alcohols **144** (Figure 13A). The position of the two double bonds at the final cyclopentadiene ring strongly depends on the size and type of the fused cycle, mostly carbocycles and *N*-heterocycles (Rinaldi et al., 2018). Bandini et al. described a related [3,3]-sigmatropic rearrangement for the site-selective gold(I)-mediated dearomatization of napthylpropynol derivatives toward dihydrofurylnaphthalen-2(1*H*)-ones (An et al., 2017).

In 2018, Nakamura et al. published the gold(I)-catalyzed intramolecular S_*E*_Ar reaction of isoxazoles **145** substituted by a propargyl amine or ether at C4 position. This cycloisomerization furnishes the fused isoxazolo substrates **146** in good yields at 60°C (Figure 13B). Moreover, the authors demonstrated that the addition of an external hydride acceptor as *N*-phenylbenzaldimine increased the yield in the synthesis of isoxazolopyridines (**146**, X = NH). Remarkably, the use of bulky gold(I)-complexes, such as [JhonPhosAu]^+^, is essential for the satisfactory formation of products (Morita et al., 2018).

Gold-catalyzed cyclizations in 1,6-enynes on indole scaffolds have also been reported in the last years (Pérez-Galán et al., 2016). Figure 13C shows a recent example in which *N*-substituted indoles **147** containing an alkene-tethered diyne cycloisomerize to eight-membered ring indoziline derivatives **148** under gold(I)-catalysis. Shi et al. described the use of bulky-phosphine gold complexes to promote, under thermal treatment (80°C), two intramolecular nucleophilic attacks at the C2 and C3 centers of the indole moiety of both activated internal alkyne groups. Interestingly, compounds **148** exhibit yellow-green fluorescence, so they may have potential applications in the field of OLEDs (Liu et al., 2018). Related gold(I)-mediated carbocyclizations of enynes involving an eight-membered ring formation have been described recently in the synthesis of dibenzo[*b,e*][1,4]diazocines (Ito et al., 2018). Likewise, gold(III) complexes have been reported to be able to promote carbocyclization processes. Thus, bis(indoyl)methyl alkynols **149** are transformed into substituted carbazoles **150** under catalysis of hydratated NaAuCl_4_ (Figure 13D). The mechanism for this transformation was found computationally to proceed via an unsual 1,2-migration of an indolylmethyl moiety as key step (Suárez et al., 2017). 1,2- And 1,3-quinazolinone fused pyrroles **152** and **154** were achieved involving a AuCl_3_-mediated cycloisomerization of alkynyl pyrroles **151** and **153** (Figure 13E). 1,2-Quinazolinones **152** are formed via an initial 1,3-R^3^ rearrangement followed by a 6-*exo-trig* cyclization involving the pendant pyrrol ring at **151**, while **153** is transformed into **154** directly through a 6-*exo-dig* cyclization onto the terminal akyne unit (Wei et al., 2018). Nevertheless, related structurally pyrroles bearing a non-terminal akyne experiments a 7-*endo-dig* cyclization rather than a 6-*exo-dig* (Kong et al., 2018).

Reactants containing a cyclopropane unit have gained considerable attention lately, and a number of remarkable transformations using this kind of substrates have been achieved through gold-chemistry. The *cis*-to-*trans* isomerization of cyclopropane rings has been experimental and computationally studied under gold(I) and gold(III) catalysis, concluding that chiral bisoxazoline-Au(III) complexes are the best catalysts for promoting this isomerization (Reiersølmoen et al., 2018).

**Figure 14:**
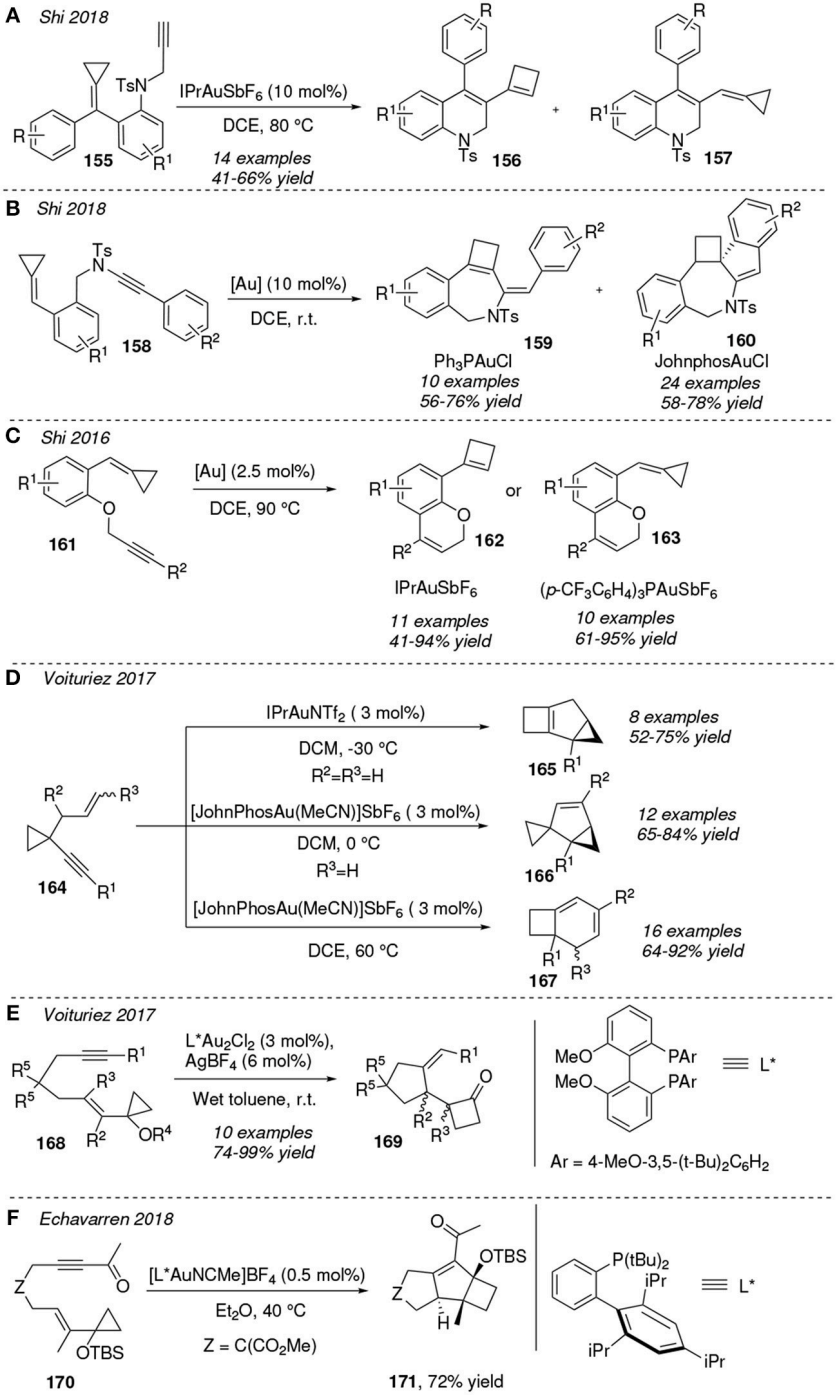
Gold-mediated cycloisomerization of 1,n-dienynes containing a cyclopropane ring.

Shi and co-workers reported a set of works on gold(I)-catalyzed cycloisomerization of enynes containing methylenecyclopropanes. The IPrAuSbF_6_ catalyst is able to activate the alkyne motif of enynes toward an intramolecular cyclization and further ring expansion of the methylenecyclopropane scaffold. For instance, aniline-linked 1,6-enynes bearing a cyclopropane ring **155** can be converted into 1,2-dihydroquinolines **156** and **157** under thermal conditions in a solution of dichloroethane. Under gold-catalysis, products **156**, containing a cyclobutene ring were the major product compared to compounds **157**, in which the cyclopropane is not expanded (Figure 14A). This product ratio is inverted when using AgSbF_6_ as catalyst (Jiang et al., 2018). This methodology was extended to methylenecyclopropane-contained ynamides **158**. Thus, substrates **158** evolve at room temperature to the polycyclic system **159** using Ph_3_PAuCl as catalyst whereas spirocyclics **160** were obtained by using a more sterically bulky catalyst, such as JohnphosAuCl. In both cases the gold-catalyst promotes the expansion of the alkylidencyclopropane to a four-member ring but only in the latter the catalyst facilities the subsequent intramolecular Friedel-Crafts process in the formation of **160** (Figure 14B) (Zhang et al., 2018b). A similar situation is reported for the intramolecular hydroarylation of the enynes **161** toward oxacycles **162** and **163** . Thus, whereas both catalysts, IPrAuSbF_6_ and (*p*-CF_3_C_6_H_4_)_3_PAuSbF_6_, promote the carbocyclization step, only the former promotes the methylenecyclopropane expansion to a cyclobutene ring (Figure 14C). The authors reasoned this different reactivity attending to the inability of the gold complex containing electron-deficient phosphine ligands to activate the alkene moiety toward the ring expansion process (Fang et al., 2016). Temperature can also be a determining factor in these reactions, as shown in the isomerization of 1,5-enynes containing a cyclopropane ring, **164**. These systems cycloisomerize to related bicyclic or spirobicyclic compounds depending on the catalyst and the thermal conditions. For instance, **164** furnishes **165** in presence of IPrAuNTf_2_ at -30°C. However, substrate **164** is converted into the biscyclopropanes **166** at 0°C under catalysis of the bulky gold complex [JohnPhosAu(MeCN)]SbF_6_ whereas bicycles **167** can be obtained at 60°C in a good diastereoisomeric ratio (Figure 14D). The authors proposed a catalytic cycle in which compounds **165** and **166** are precursors of **167** (Chen et al., 2016b). In the same line, Voituriez et al. described the enantioselective synthesis of cyclobutanone derivatives **169** via gold(I)-catalyzed cycloisomerization of enyne-methylenecyclopropanes **168**. The most broadly tested catalyst was a binuclear gold complex bearing a bulky chiral-phosphine ligand, as represented in Figure 14E. The major isolated products are those with substituents R^2^ and R^3^ presenting *cis* configuration (Wu et al., 2017). A similar approach was used as the key step in the total synthesis of the sesquiterpene Repraesentin F. Echavarren et al. reported a highly diastereoselective gold(I)-mediated cyclization of the 1,6-enyne **170** via a tandem cycloisomerization/Prins-type reaction furnishing the tricyclic systems **171** containing the unsual skeleton of this natural product. Through this protocol the diasteroisomer with the desired *anti* ring fusion configuration is obtained as major product in a 7.2:1 ratio with respect to that with the *syn* fusion (Figure 14F) (Ferrer and Echavarren, 2018b).

**Figure 15:**
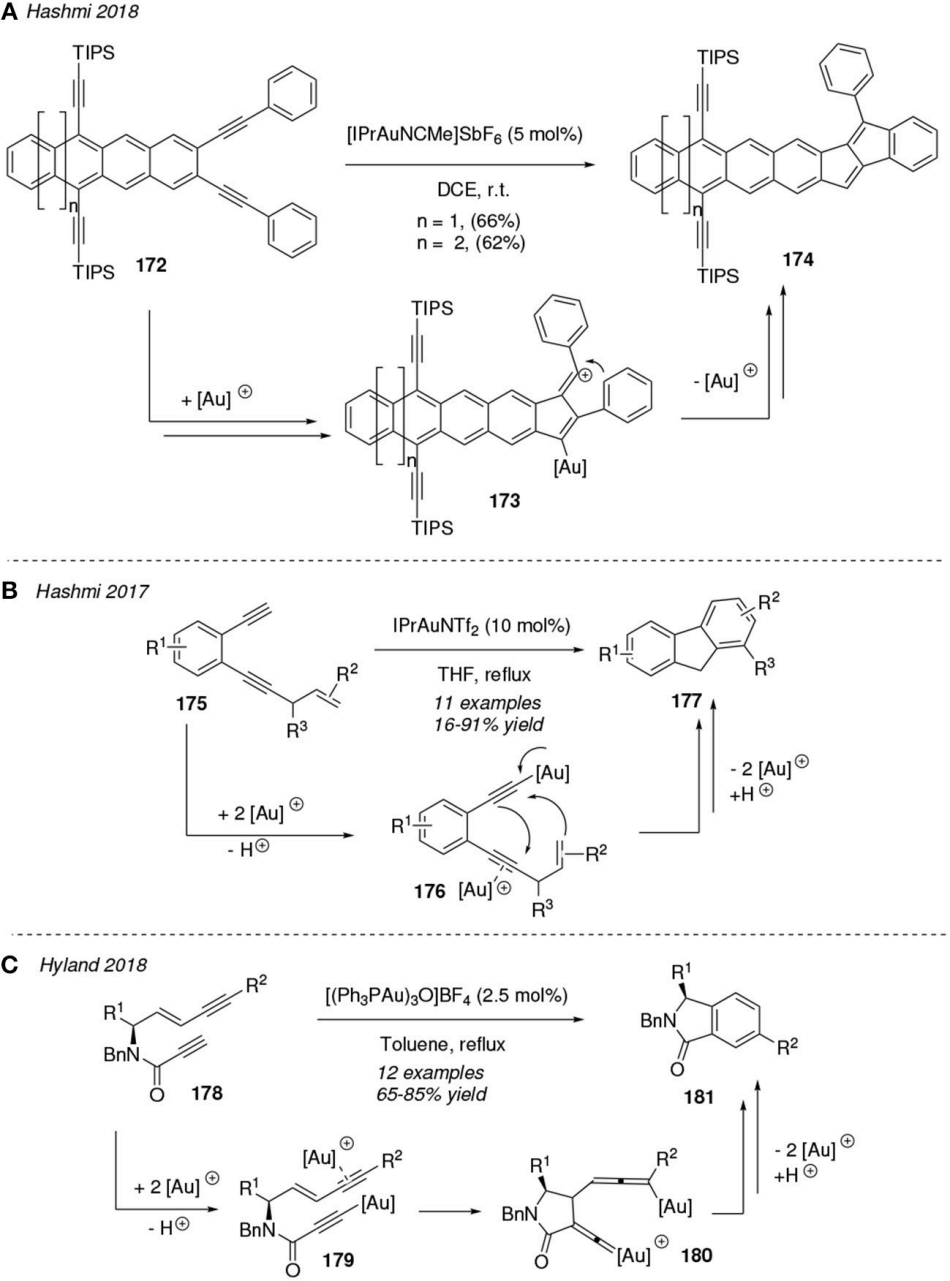
Gold-mediated cycloisomerization reactions of 1,*n*-diynes.

The reactivity pattern of 1,*n*-diynes has also been smartly exploited toward the construction of complex highly π-conjugated polycyclic systems (Asiri and Hashmi, 2016). In this sense, the works from Hashmi stand out with several remarkable publications on this topic in the last years (Tšupova et al., 2017; Wurm et al., 2017, 2018). A representative example is shown in Figure 15A. They described the gold-mediated cycloisomerization of 1,5-diynes building on an aromatic ring **172** toward aromatic polycyclic-annulated systems **174**. The reaction mechanism involves a vinyl cation intermediate **173** and operates under mild reaction conditions. Interestingly optical properties have been described in this kind of annulated-pentalene compounds making them potential candidates for future optoelectronic devices (Sekine et al., 2018). Another example by Hashmi et al. was reported in 2017 and was highlighted as the first intramolecular trapping of dually gold-activated intermediates **176** with an olefinic C(sp^2^)-H bond (Figure 15B). 1,5-Diynes bearing an allyl-substituted alkene attached to an aromatic skeleton (**175**) could also be converted into fluorene derivatives **177** via a dehydrogenative dual gold-catalyzed activation at reflux of tetrahydrofuran (Bucher et al., 2017). In this sense, dual-gold catalysis has been implemented in the cycloaromatization of unconjugated *(E)*-enedynes **178** toward isoindolines **181** by using the trigold oxo complex [(Ph_3_PAu)_3_O]BF_4_ as catalyst (Figure 15C). Both specific experiments with deuterated reactants and a detailed theoretical study indicate that the most feasible mechanistic pathway is that involving the very reactive allenyl-gold/gold-vinylidene intermediate **180**, which is generated from a dual-gold activated substrate **179** via a 5-*exo*-trig cyclization. The subsequent carbocyclization and rearomatization afforded the enantioriched isoindolines **181** in good yields (Zamani et al., 2019).

## 4. Conclusions

In this review, the interest on gold and its relevance in intramolecular isomerization reactions is confirmed by taking into account the vast number of studied reported in the last 3 years and covered here. Most of the isomerization processes included in this review are initiated by three main different kind of transformations at allene and alkyne sites: nucleophilick attack onto the activated alkyne, 1,3 or 1,2- rearrangement of a carbonate/ester group over the π-system or a carbocyclization process. As we have shown, structurally complex molecules can be synthesize from easily reachable reactants under gold-catalysis and mild reaction conditions. Fewer in number but still important, some studies have developed gold(III)-catalysts although gold(I)-complexes seem to be more versatile, effective and preferred in this kind of reactions. Nowadays, enantioselective synthesis is a paramount goal, particularly when chemists are involved in biologically relevant molecules. Accordingly, the number of studies involving gold-complexes containing chiral ligands is growing notably. Although we can consider gold as a “young” metal, chemically speaking, it is clear that this metal center has many advantages to take into account when designing and performing innovative, complexity oriented and efficient chemical transformations.

## Author Contributions

CS, ON, and MM-L contributed conception of the review article, scope and structure. MM-L drafted the article. CS and ON revised its scientific and formal content.

### Conflict of Interest Statement

The authors declare that the research was conducted in the absence of any commercial or financial relationships that could be construed as a potential conflict of interest.

## Footnotes

^1^The Scopus database returns 30,000 entries when looking for *gold catalyst, gold cataysis or gold-mediated* keywords (search performed in January 2019)
